# Identification and Tissue-Specific Characterization of Novel SHOX-Regulated Genes in Zebrafish Highlights SOX Family Members Among Other Genes

**DOI:** 10.3389/fgene.2021.688808

**Published:** 2021-05-27

**Authors:** Sandra Hoffmann, Ralph Roeth, Sabrina Diebold, Jasmin Gogel, David Hassel, Steffen Just, Gudrun A. Rappold

**Affiliations:** ^1^Department of Human Molecular Genetics, Institute of Human Genetics, University Hospital Heidelberg, Heidelberg, Germany; ^2^DZHK (German Centre for Cardiovascular Research), Partner Site Heidelberg/Mannheim, Heidelberg, Germany; ^3^nCounter Core Facility, Institute of Human Genetics, University of Heidelberg, Heidelberg, Germany; ^4^Clinic for Internal Medicine II - Molecular Cardiology, University Hospital Ulm, Ulm, Germany; ^5^Department of Internal Medicine III ‐ Cardiology, University Hospital Heidelberg, Heidelberg, Germany

**Keywords:** short stature, skeletal dysplasia, pectoral fins, zebrafish, *SHOX* deficiency, skeletal disease associations

## Abstract

*SHOX* deficiency causes a spectrum of clinical phenotypes related to skeletal dysplasia and short stature, including Léri-Weill dyschondrosteosis, Langer mesomelic dysplasia, Turner syndrome, and idiopathic short stature. SHOX controls chondrocyte proliferation and differentiation, bone maturation, and cellular growth arrest and apoptosis *via* transcriptional regulation of its direct target genes *NPPB*, *FGFR3*, and *CTGF*. However, our understanding of SHOX-related pathways is still incomplete. To elucidate the underlying molecular mechanisms and to better understand the broad phenotypic spectrum of *SHOX* deficiency, we aimed to identify novel SHOX targets. We analyzed differentially expressed genes in *SHOX*-overexpressing human fibroblasts (NHDF), and confirmed the known SHOX target genes *NPPB* and *FGFR* among the most strongly regulated genes, together with 143 novel candidates. Altogether, 23 genes were selected for further validation, first by whole-body characterization in developing *shox*-deficient zebrafish embryos, followed by tissue-specific expression analysis in three *shox*-expressing zebrafish tissues: head (including brain, pharyngeal arches, eye, and olfactory epithelium), heart, and pectoral fins. Most genes were physiologically relevant in the pectoral fins, while only few genes were also significantly regulated in head and heart tissue. Interestingly, multiple *sox* family members (*sox5*, *sox6*, *sox8*, and *sox18*) were significantly dysregulated in *shox*-deficient pectoral fins together with other genes (*nppa*, *nppc*, *cdkn1a*, *cdkn1ca*, *cyp26b1*, and *cy26c1*), highlighting an important role for these genes in *shox*-related growth disorders. Network-based analysis integrating data from the Ingenuity pathways revealed that most of these genes act in a common network. Our results provide novel insights into the genetic pathways and molecular events leading to the clinical manifestation of *SHOX* deficiency.

## Introduction

Linear growth and skeletal development are highly dynamic processes that depend on transcription factors, signaling molecules, and extracellular matrix proteins. Long bones grow by endochondral ossification, a sequential replacement of cartilaginous tissue by bone. The growth plate, located near the ends of the long bones, provides a continuous supply of chondrocytes for endochondral ossification, which is initiated during early fetal development and continues until adolescence (Kronenberg, 2003; Lui et al., 2018). Any failure in this process causes a wide range of skeletal disorders. SHOX is one of several critical factors regulating chondrogenesis in the growth plate (Marchini et al., 2016).

Alterations in *SHOX* expression impairs human growth and causes a spectrum of clinical phenotypes related to skeletal dysplasia and short stature including Léri-Weill dyschondrosteosis, Langer mesomelic dysplasia, Turner syndrome, and idiopathic short and tall stature (Rao et al., 1997; Belin et al., 1998; Shears et al., 1998; Oliveira and Alves, 2011; Fukami et al., 2016; Marchini et al., 2016; Monzani et al., 2019). However, no genotype-phenotype correlation exists in individuals with *SHOX* mutation/deletion within the coding region (Binder et al., 2004), and also downstream enhancer deletions do not seem to greatly differ from those with coding mutations/deletions with respect to phenotypic characteristics and severity (Benito-Sanz et al., 2005; Rosilio et al., 2012). Although the penetrance of *SHOX* deficiency is high, its clinical expression is variable, even among family members (Schiller et al., 2000; Binder, 2011). *CYP26C1* has been identified as a modifier of severity in patients with *SHOX* deficiency, but certainly others will follow that contribute to the variable expressivity (Montalbano et al., 2016).

SHOX is a transcriptional regulator in chondrocyte proliferation and differentiation, bone maturation, cartilage synthesis, and cellular growth arrest and apoptosis *via* its direct target genes *NPPB*, *FGFR3*, and *CTGF* (Marchini et al., 2004, 2007; Decker et al., 2011; Beiser et al., 2014; Hristov et al., 2014). It also promotes linear growth at the growth plate *via* interaction with the SOX trio (SOX5, SOX6, and SOX9; Aza-Carmona et al., 2011). Further characterization of SHOX-dependent networks is critical, not only for elucidating SHOX functions and their link to disease, but also for discovering novel therapeutic targets in SHOX-related disorders.

To better understand the disease mechanisms underlying *SHOX* deficiency, we first performed genome-wide expression profiling in *SHOX*-overexpressing human fibroblasts to uncover novel SHOX target genes. Considering the complexity of the growth plate and the spatiotemporal expression of SHOX during bone development, we aimed to validate the physiological relevance of the identified genes in an animal model.

A *SHOX* ortholog is not present in rodents, so zebrafish has been a valuable model for studying the role of SHOX during development. Zebrafish embryos are excellent models for studying bone physiology and pathology (Carnovali et al., 2019) because they are small, have rapid external development, have a transparent larval body, and can easily be manipulated genetically. Another important consideration is that a *shox* ortholog and conserved non-coding elements (CNEs) are present in the zebrafish genome (Kenyon et al., 2011; Verdin et al., 2015). Several studies have already confirmed that *shox* deficiency impairs early embryonic growth and bone formation in zebrafish and that *shox* morphants have a shorter body length and reduced pectoral fin size (Sawada et al., 2015; Montalbano et al., 2016; Yokokura et al., 2017). Therefore, we used zebrafish as a read-out animal model to validate our putative target genes and confirm their physiological relevance. Our study highlights the importance of various gene families in *shox*-related growth disorders including the cyclin-dependent kinase inhibitors (*CKI*), cytochrome P450 26 subfamily (*CYP26*), natriuretic peptides (*NP*), and SRY-related HMG-box (*SOX*) genes.

## Materials and Methods

### Cell Culture

Normal human dermal fibroblasts (NHDF cells; PromoCell: C12300) were cultured in Dulbecco’s Modified Eagle Medium (DMEM; Gibco, Thermo Fisher Scientific), supplemented with 10% fetal bovine serum, penicillin (100 U/ml), and streptomycin (100 μg/ml) in a 5% CO_2_-humidified incubator at 37°C. 1 × 10^6^ NHDF cells were transfected with 3.5 μg pcDNA4/TO-*SHOX*-WT using the Amaxa® Human Dermal Fibroblasts Nucleofector® Kit (Lonza) according to the manufacturer’s instructions. In control experiments, NHDF were transfected with 3.5 μg pcDNA4/TO-*SHOX*-HM, which encodes a homeodomain-mutant of SHOX leading to the amino acid exchange Y141D.

### Microarray Analysis

Gene expression profiling was performed using the Sentrix® HumanRef-8 Expression BeadChips from Illumina according to the manufacturer’s protocol. Total RNA from NHDF cells was isolated using Illustra RNAspin Isolation Kit (Cytiva) according to the manufacturer’s instructions. For array hybridization, we prepared biotin-labeled cRNA from 250 μg total RNA according to Illumina’s recommended sample labeling procedure using the MessageAmp™ II aRNA Amplification Kit (Thermo Fisher Scientific). Hybridization was performed at 58°C in GEX-HCB buffer (Illumina) at a concentration of 100 ng cRNA/μl for 20 h. Microarrays were washed twice in E1BC buffer (Illumina) at room temperature for 5 min. After blocking for 5 min in 1% Blocker Casein in PBS, array signals were developed by a 10 min incubation in 1 μg/ml Cy3-streptavidin (Cytiva) solution and 1% blocking solution. After final wash in E1BC, the arrays were dried and scanned using a Beadstation array scanner. Data extraction was done for all beads individually, and outliers were removed if >2.5 median absolute deviation (MAD). All remaining data points were used for the calculation of the mean average signal and SD for each probe. For each analyzed time point, we compared signals of normalized gene expression of *SHOX*-WT transfected samples to *SHOX*-HM transfected controls. Differentially expressed genes were defined by a change in expression of more than 3-fold (*SHOX*-WT/*SHOX*-HM).

### Zebrafish Embryos and Microinjections

Zebrafish (*Danio rerio*) were maintained as previously described (Westerfield, 2000). For all morpholino injection procedures, the Tg (myl7:GFP) strain was used (Rottbauer et al., 2006). Morpholino-modified antisense oligonucleotides (MO; Gene Tools) were directed against the exon 2 intron 2 junction in *shox*, causing reduction of pectoral fins as described previously (Montalbano et al., 2016). *Shox* antisense oligonucleotides or a standard control oligonucleotide (MO control), diluted in 0.2 M KCl, were microinjected into one-cell-stage zebrafish embryos (Just et al., 2011).

### nCounter Expression Analysis

Zebrafish tissues were isolated at 55 hours post-fertilization (hpf) and total RNA was extracted with the Direct-zol RNA Microprep Kit (Zymo Research) according to the manufacturer’s instructions. For each experiment, whole zebrafish embryos (10–15), hearts (30–70), heads (~20), or pectoral fins (30–40) were pooled per condition to obtain 50 ng of input material. Three to five independent experiments (*n*) were performed. mRNA expression levels were measured at the nCounter Core Facility Heidelberg using the nCounter SPRINT Profiler. This RNA quantification technology utilizes a direct digital detection of mRNA molecules and allows multiplexed target measurement in a single reaction with high sensitivity and specificity even with low amounts of input material (50 ng). A detailed probe design is given in Supplementary Table 1. The workflow is described at http://www.nanostring.com/elements/workflow. Background correction and data normalization were performed using the nSolver Analysis Software 4.0 (NanoString Technologies). The most stably expressed genes were chosen for normalization based on the geNorm method.

### Statistical Analyses

Statistical analysis was carried out using GraphPad Prism 9 (GraphPad Software, La Jolla, CA, United States). For multiple *t*-tests, the two-stage step-up method of Benjamini, Krieger, and Yekutieli with a desired false discovery rate (FDR) of 5% was used to correct the values of *p*. All conditions statistically different from the control were indicated by ^*^p < 0.05, ^**^p < 0.01, and ^***^p < 0.001.

### Ingenuity Pathway Analysis

Ingenuity pathway analysis (IPA) is a web-based software application for the evaluation, integration, and interpretation of omics data (Kramer et al., 2014; QIAGEN Inc.).^1^ IPA analysis indicates relationships and interactions between genes based on published data. A list of 24 selected genes (including *SHOX*) was uploaded to perform a pathway and core analysis. The core analysis comprises enrichment pathway analysis to identify significant biological processes and molecular functions in which these genes are involved. These include, for example, differentiation and development of chondrocytes, cartilage and connective tissue, as well as growth failure, short stature, and limb defects.

### Databases

DisGeNET database (Pinero et al., 2015).^2^ This database enables browsing, searching, and analyzing of genetic information linked to human diseases based on expert curated repositories, GWAS catalogues, animal models, and the scientific literature. DisGeNET is a discovery platform containing one of the largest publicly available collections of genes and variants associated to human diseases. The current version of DisGeNET (v7.0) contains 1,134,942 gene-disease associations, between 21,671 genes and 30,170 diseases, disorders, traits, and clinical or abnormal human phenotypes, and 369,554 variant-disease associations, between 194,515 variants and 14,155 diseases, traits, and phenotypes.

## Results

*SHOX* expression is low in tissues and cell lines (Durand et al., 2011) and only detectable at a very low level in mammalian organs during development (Supplementary Figure 1). The highest endogenous expression of *SHOX* so far was found in primary human fibroblasts (NHDF; Durand et al., 2011) and zebrafish embryos (Sawada et al., 2015; Montalbano et al., 2016). But even in fibroblasts, expression levels are too low to perform reliable knockdown experiments.

To investigate SHOX regulatory pathways and identify novel SHOX targets, we analyzed transcriptional changes following overexpression of *SHOX* wild type (WT) compared with a functional homeodomain mutant (HM) in NHDF fibroblast cells. *SHOX*-HM was previously detected in a patient with short stature carrying a c.421T>G missense mutation that led to an amino acid exchange (p.Y141D) in the homeodomain, which impairs SHOX function by reducing DNA-binding and transactivation capacity (Schneider et al., 2005). Therefore, this variant is an ideal negative control for differential expression analysis.

**Figure 1:**
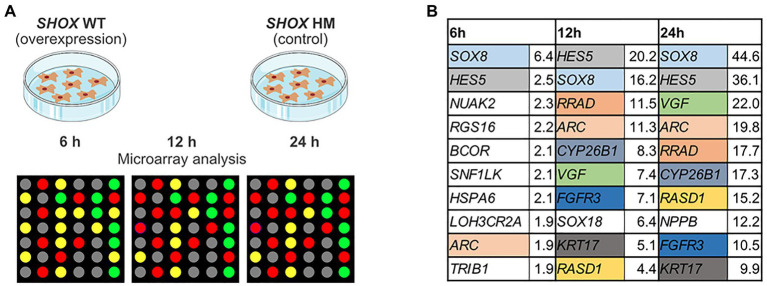
Microarray analysis of *SHOX* overexpressing wild type (WT) and mutant (HM) normal human dermal fibroblast (NHDF) cells. **(A)** Overview of experimental design. **(B)** Top 10 regulated genes in *SHOX* overexpressing NHDF cells after 6, 12, and 24 h. Identical colors indicate the same genes at different time points. Relative expression ratios are indicated next to the gene’s name.

Gene expression profiling was carried out in NHDF cells at three different stages: 6, 12, and 24 h after transfection (Figure 1A). Whole-genome Illumina HumanRef8 expression beadchips (Human Sentrix-8 V2) were used to measure gene expression at each time point. Normalized expression of *SHOX*-WT was compared with normalized expression of *SHOX*-HM in the cells and displayed as ratios (Supplementary Tables 2–4). Microarray data of all three time points were then merged to analyze all differentially expressed genes (Supplementary Table 5). Out of 24,500 annotated transcripts targeted by the Illumina expression beadchips, 145 genes were differentially expressed with a more than 3-fold change. Forty-eight genes were downregulated and 97 genes were upregulated upon *SHOX* overexpression. The top 10 upregulated genes at all three time points are shown in Figure 1B. At the earliest time point (6 h), only one gene (*SOX8*) was significantly upregulated after *SHOX* overexpression based on a threshold 3-fold change in expression. At 12 and 24 h after transfection, the top 10 differentially expressed genes were all upregulated more than 3-fold (Figure 1B).

We identified two known SHOX target genes in the top 10 list – *NPPB* (Marchini et al., 2007) and *FGFR3* (Decker et al., 2011). In addition, we found novel target genes from families already associated with SHOX (Supplementary Table 6). These included *SOX8* and *SOX18* of the *SOX* gene family and *CYP26B1* of the *CYP26* gene family. *SOX18*, which was in the top 10 list upon 12 h of *SHOX* expression, fell to position 11 after 24 h despite a 9-fold upregulation. *NPPB* only appeared in the top 10 list after 24 h of *SHOX* expression. To further investigate family-based regulations, we selected further gene members (*n* = 4) of these families for validation, as well as eight genes that were previously associated with SHOX in the literature. The 11 genes from our list of top-regulated genes (Figure 1B) and the additional 12 genes from the literature are summarized in Table 1.

**Table 1:** Genes selected for validation.

Gene	6 h	12 h	24 h
*ARC*	1.9	11.3	19.8
*BMP4*	1	1	1
*CDKN1A*	1.2	2.9	5.4
*CDKN1B*	1.4	0.7	1
*CDKN1C*	1.1	1.8	5.1
*CYP26A1*	1.1	1.1	1
*CYP26B1*	1	8.3	17.3
*CYP26C1*	1	1.1	1
*FGFR3*	1.7	7.1	10.5
*HES5*	2.5	20.2	36.1
*KRT17*	1.4	5.1	9.9
*NPPA*	1	1.1	1.4
*NPPB*	1	1.7	12.2
*NPPC*	1	0.9	1
*RASD1*	1.5	4.4	15.2
*RRAD*	1	11.5	17.7
*SHOX2*	1	1	1
*SOX5*	1	1	1
*SOX6*	1.2	1	1
*SOX8*	6.4	16.2	44.6
*SOX9*	1	0.8	0.8
*SOX18*	1.1	6.4	9.0
*VGF*	1.1	7.4	22.0

The selection is based on the top regulated genes in SHOX overexpressing NHDF cells after 12 and 24 h (blue; *n* = 11). Already known SHOX-associated genes are highlighted in green (*n* = 8) and additionally analyzed members of gene families in light gray (*n* = 4).

**Figure 2:**
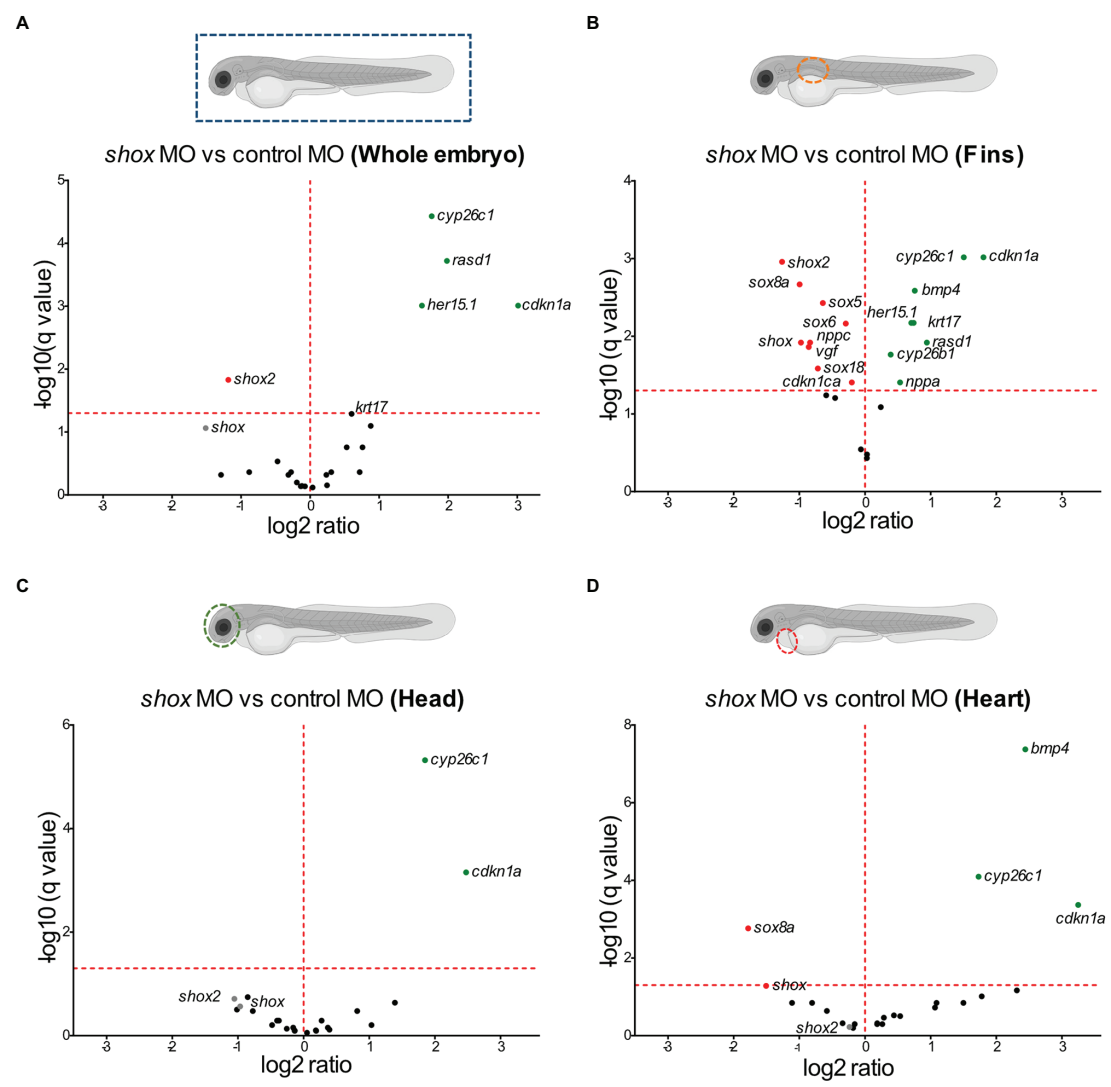
Tissue-specific validation of putative Shox target genes in zebrafish embryos after *shox* knockdown. Volcano plot presentations of expression ratio of selected shox target genes in **(A)** whole zebrafish tissue (*n* = 6 *shox* MO, *n* = 6 control MO), **(B)** fins (*n* = 3 *shox* MO, *n* = 3 control MO), **(C)** head (*n* = 4 *shox* MO, *n* = 4 control MO), and **(D)** heart (*n* = 4 *shox* MO, *n* = 5 control MO) at 55 hours post-fertilization (hpf) after morpholino-mediated knockdown of shox compared to controls. Genes above the red horizontal dashed line are significantly regulated. Genes shown to the left of the dashed vertical line are downregulated, those on the right are upregulated. Statistical significance was determined by multiple unpaired *t*-tests adjusted by the two-stage step-up method of Benjamini, Krieger, and Yekutieli (adjusted values of *p* = *q*-values). MO, morpholino.

We used zebrafish embryos as an *in vivo* model to verify our selected putative SHOX-regulated genes. The expression of the zebrafish orthologs (*n* = 22; *ARC* does not have an ortholog) was analyzed at 55 hpf after morpholino-mediated knockdown of *shox*. Differential expression was found for 5/22 analyzed genes (*shox2*, *her15.1*, *cdkn1a*, *rasd1*, and *cyp26c1*) after *shox* knockdown using RNA isolated from whole zebrafish embryos (Figure 2A). Knockdown efficiency was only nominally significant for *shox* (*p* = 0.0284; *q* = 0.0635). To provide a possible explanation for this, we addressed its tissue-specific regulations. Thus, we performed analyses in *shox*-expressing zebrafish tissues from three different regions: pectoral fins, head (brain, pharyngeal arches, eye, and olfactory epithelium), and heart of *shox*-deficient and control embryos at 55 hpf (Figures 2B–D).

**Figure 3:**
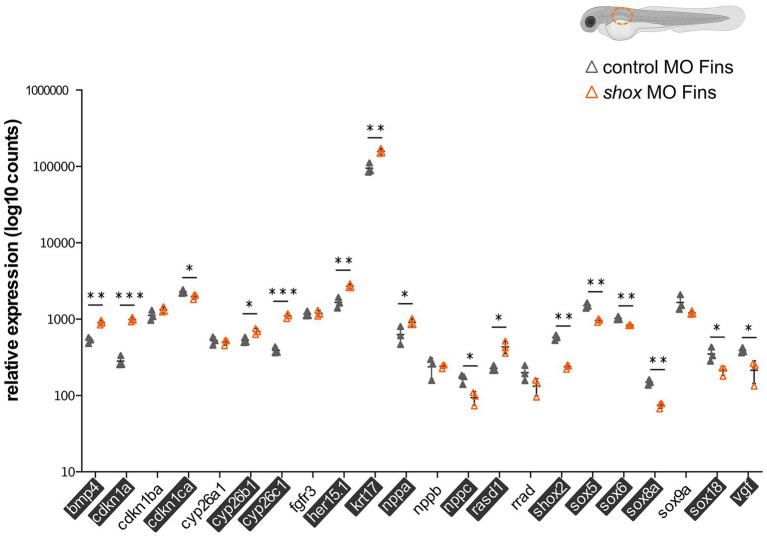
Validation of putative Shox target genes in zebrafish fins. Tissue-specific analysis of all selected shox targets upon morpholino-mediated *shox* knockdown in zebrafish fins 55 hpf (orange) compared to controls (gray); *n* = 3 experiments per condition. Significantly regulated genes are highlighted. For *ARC*, no Zebrafish otholog exists. *HES5* corresponds to *her15.1* in zebrafish. Statistical significance was determined by multiple unpaired *t*-tests adjusted by the two-stage step-up method of Benjamini, Krieger, and Yekutieli (adjusted values of *p* are indicated by ^*^p < 0.05; ^**^p < 0.01; and ^***^p < 0.001). MO, morpholino; hpf, hours post-fertilization.

**Figure 4:**
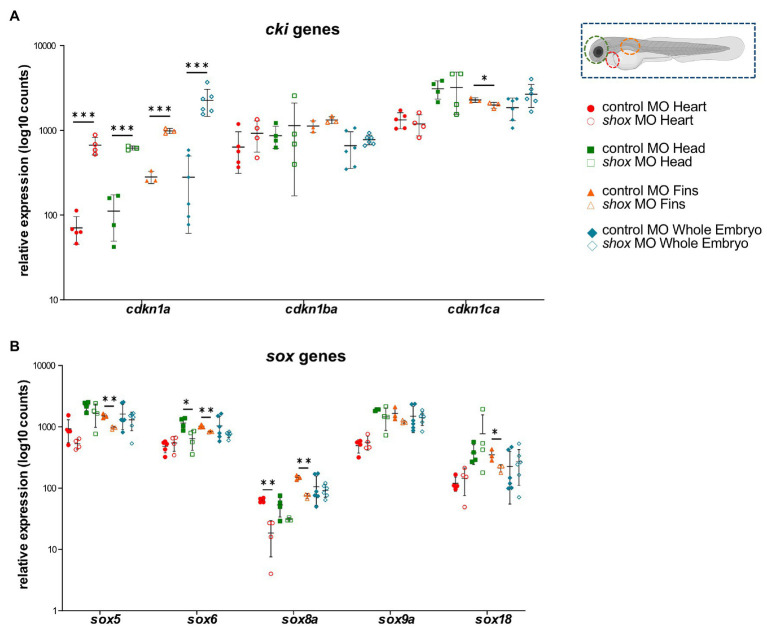
Tissue-specific analysis of gene families upon morpholino-mediated *shox* knockdown in zebrafish embryos 55 hpf compared to controls. Relative expression levels for **(A)** the cyclin-dependent kinase inhibitor (*cki*) family members *cdkn1a*, *cdkn1ba*, and *cdkn1ca*; **(B)** members of the SRY-related HMG-box (*sox*) genes *sox5*, *sox6*, *sox8a*, *sox9a*, and *sox18* in heart (red; *n* = 4–5), head (green; *n* = 4), fin (orange; *n* = 3) or whole embryo (blue; *n* = 6). Statistical significance was determined by multiple unpaired *t*-tests adjusted by the two-stage step-up method of Benjamini, Krieger, and Yekutieli (adjusted values of *p* are indicated as ^*^p < 0.05, ^**^p < 0.01, and ^***^p < 0.001). MO, morpholino.

After *shox* knockdown, *shox* was significantly downregulated in the pectoral fin (*p* = 0.0149; *q* = 0.0118) but not in the head (*p* = 0.0648; *q* = 0.2381), and nominally significant downregulation was detected in the heart (*p* = 0.0142; *q* = 0.0566) as measured by nCounter analysis (Figures 2B–D). Almost all analyzed genes (16/22) were significantly dysregulated in the pectoral fins at the developmental time point 55 hpf (Figure 3). Of the top 10 regulated genes in NHDF cells, seven were dysregulated in zebrafish fins (*cyp26b1*, *her15.1*, *krt17*, *rasd1*, *sox8a*, *sox18*, and *vgf*), although the regulatory direction was not always the same as in NHDF cells. Interestingly, members of the *sox* and *cki* family were significantly dysregulated in the pectoral fin (Figure 4), highlighting the important role of these gene families in Shox-related growth defects.

**Figure 5:**
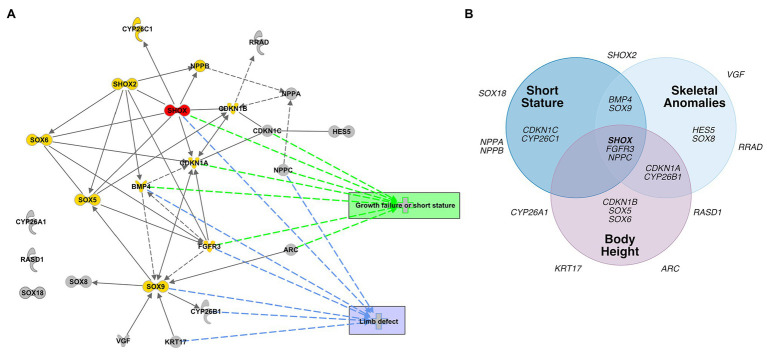
Database analyses of SHOX-regulated genes. **(A)** Network-based pathway analysis of SHOX-regulated genes using the Ingenuity pathway analysis (IPA) software. SHOX is highlighted in red. Known SHOX-associated genes are shown in yellow. Novel SHOX-regulated genes are shown in gray. Direct regulations are indicated by arrows and interactions as lines; indirect regulations or interactions are presented as dotted arrows/lines, respectively. The most important annotated pathways relevant to SHOX-associated diseases or function are highlighted in green (genes involved in growth failure and short stature) and blue (genes involved in limb defects). **(B)** Gene-disease association according to DisGeNET. Venn diagram showing associations between SHOX and 23 SHOX-regulated genes with respect to skeletal phenotypes based on the DisGeNET database (https://www.disgenet.org/home/; Pinero et al., 2015).

To identify transcriptional pathways in which the novel SHOX-dependent genes play a role, we performed a functional network analysis, integrating the 23 selected genes with SHOX using the IPA software tool. Our analysis demonstrates that the majority of the newly identified/selected genes are connected within the same network of the known SHOX-regulated genes (except *CYP26A1*, *RASD1*, and *SOX18*). We also identified significant biological processes and molecular functions that involve these genes (Supplementary Table 7). These included differentiation and development of chondrocytes, cartilage, and connective tissue. The most important annotated pathways relevant to SHOX-associated diseases included genes involved in growth failure, short stature, and limb defects (Figure 5A).

To predict the pathological relevance of the SHOX-regulated genes, we used the DisGeNET database (see footnote 2; Pinero et al., 2015) to summarize the known disease associations for each of the selected 23 genes (Figure 5B). Thirteen genes were associated with skeletal phenotypes such as short stature, skeletal abnormalities, and height; 10 genes had no such associations to date and, therefore, might represent novel candidates for skeletal disease.

## Discussion

Identifying SHOX-regulated genes is essential to understanding how SHOX signaling exerts its diverse effects in individuals with *SHOX* deficiency. To identify novel target genes, we compared the transcriptional activity of wild type SHOX with a non-functional mutant form of SHOX in NHDF cells by microarray analysis. Although derived from human fibroblasts, NHDF cells do not fully reflect the situation in the growth plate. Despite this limitation, all identified 145 differentially up‐ and downregulated target genes downstream of SHOX can now be further investigated as candidates for various disorders. The proteins encoded by this list of SHOX-regulated genes are implicated in a wide variety of functions, consistent with the diverse effects of SHOX on development (Marchini et al., 2016). These functions include chondrocyte proliferation and differentiation, bone maturation, cartilage synthesis, cellular growth arrest, and apoptosis. Some genes represent known direct targets or have been previously associated with SHOX, but most are unknown SHOX-regulated genes. The known SHOX target genes *NPPB* and *FGFR3* (Marchini et al., 2007; Decker et al., 2011) were among the top regulated genes after 12 and 24 h of overexpression. These findings show that NHDF cells are a valid and valuable model for analyzing the gene networks regulated by SHOX *in vitro*.

Considering the complexity of bone development and the spatiotemporal expression of *SHOX*, 11 of the upregulated and 12 additional genes were chosen for *in vivo* validation in zebrafish. Previous evidence has highlighted the role of SHOX as a transcriptional activator (Rao et al., 2001); therefore, we focused in this study only on those transcripts that were upregulated in NHDF cells and selected these for validation. *Shox* knockdown in whole zebrafish embryos altered embryonic growth and bone formation as previously described (Sawada et al., 2015; Montalbano et al., 2016; Yokokura et al., 2017). Gene expression analysis in *shox*-deficient whole zebrafish embryos also demonstrated a significant downregulation of *shox2*, the only *shox* paralog, and upregulation of four selected target candidates (*cyp26c1*, *rasd1*, *her15.1*, and *cdkn1a*). A *shox2*-associated cardiac phenotype is not present in whole embryos (Blaschke et al., 2007; Hoffmann et al., 2013), despite its strong downregulation (*p* = 0.0029; *q* = 0.0010) and known role in the orchestration of heart function.

To uncover any tissue-specific differences in SHOX-mediated gene expression, we performed separate analyses in fin, heart, and head tissue. Some genes (e.g., *shox2*) were downregulated in fin and head tissue after *shox* knockdown but not in heart tissue. This explains why we did not observe a cardiac phenotype in these embryos (Figure 2). *Bmp4* and *sox8a* were significantly dysregulated in fin and heart but not in whole zebrafish embryos, indicating the importance of tissue-specific analyses to identify cell-specific effects that may not be detected in whole embryos.

A more specific investigation, particularly in the fins, provided further insight: indeed, 16 of the 22 analyzed target candidates revealed significantly differential expression in zebrafish fins. The most downregulated gene was *shox2*, while *cdkn1a* was the most upregulated one. SHOX and SHOX2 proteins are known to interact (Aza-Carmona et al., 2014), but this is the first published evidence that *shox2* is a downstream target of Shox in the zebrafish fin. *Cdkn1a*, on the other hand, is a known cellular mediator of SHOX and is upregulated in *SHOX*-expressing cells (Marchini et al., 2004); however, we observed an upregulation of *cdkn1a* after *shox* knockdown in zebrafish fins. The known SHOX targets *NPPB* and *FGFR3* were dysregulated in NHDF cells but not in zebrafish. Different directions of regulation in different model systems have been observed for SHOX targets. SHOX was shown to positively regulate *FGFR3* expression in NHDF cells, confirming our results, but *Shox* overexpression in limb bud-derived chicken micromass cultures resulted in downregulation of *Fgfr3* and may explain the almost mutually exclusive expression patterns of *Fgfr3* and *Shox* in embryonic chicken limbs (Decker et al., 2011). Tissue-specific and cofactor-dependent opposing regulation of genes by specific transcription factors has been reported before (Lambert et al., 2018). Cofactors (coactivator or corepressor) vary in their availability and interaction with transcription factors in different tissues and this variation mediates distinct cell-specific effects. For example, *Bmp4* is a direct transcriptional target of Shox2 and is positively regulated during cardiogenesis, whereas an opposite regulation has been described in limb development (Yu et al., 2007; Puskaric et al., 2010; Bobick and Cobb, 2012). This may explain why some genes that are upregulated after *SHOX* overexpression in NHDF cells are not downregulated after *shox* knockdown in zebrafish fins. Yet, differential expression was identified for many genes in both zebrafish and human cells, indicating conserved regulatory mechanisms between these two species.

Multiple members of the sex determining region Y-box (*sox*) family (*sox5*, *sox6*, *sox8a*, and *sox18*) were shown to be significantly dysregulated in *shox*-deficient pectoral fins among other genes (*nppa*, *nppc*, *cdkn1a*, *cdkn1ca*, *cyp26b1*, and *cy26c1*), highlighting the important role of various gene family members in Shox-related growth disorders. SHOX is known to interact with the SOX trio (SOX5/SOX6 and SOX9; Aza-Carmona et al., 2011), but we show for the first time that Shox transcriptionally regulates *sox5* and *sox6* but not *sox9*. Knockdown of *Sox6* downregulates *Shox* expression in chicken (Lin et al., 2018), suggesting a reciprocal regulation between *shox* and *sox6*. Two other SOX family members, *SOX8* and *SOX18*, were among the most upregulated genes in NHDF cells and confirmed to be significantly downregulated in *shox*-deficient pectoral fins. Among the SOX proteins, SOX9 exhibits the closest similarity to SOX8 (Schepers et al., 2000), but the role of SOX8 during chondrogenesis is not completely understood. *SOX8* is highly upregulated during chondrogenesis *in vitro*, most likely under the control of SOX9 and both together with SOX5 and SOX6 are required for transcription of *COL2A1* and many other chondrogenic molecules (Herlofsen et al., 2014). Thus, *SOX8/sox8a* might promote chondrogenesis *via* a *SHOX/shox* network. *SOX18* silencing significantly induces cell cycle arrest and apoptosis, and inhibits cell migration, invasion, and cell growth *in vitro* and *in vivo*. In contrast, *SOX18* is overexpressed in osteosarcoma, and promotes cell proliferation, migration, and invasion, inhibiting cell cycle arrest and apoptosis (Zhu et al., 2018). Given the role of SHOX as a modulator of cell proliferation and apoptosis (Marchini et al., 2004; Hristov et al., 2014), shox-dependent regulation of *sox18* might play a role in these processes.

Other interesting gene family members regulated by Shox are the cyclin-dependent kinase inhibitor 1 (*cki*) genes. Two members, *CDKN1A* and *CDKN1B*, have previously been reported as cellular mediators of SHOX. In human osteosarcoma cells, the induction of *SHOX* expression elevates *CDKN1A* and *CDKN1B* levels (Marchini et al., 2004). Together with *CDKN1C*, this gene family contributes to the regulation of cell cycle progression by regulating the activity of cell cycle kinases during chondrogenic differentiation (Yan et al., 1997; Negishi et al., 2001; Bellosta et al., 2003; Rentsendorj et al., 2010; Cardelli et al., 2013). In *shox*-deficient zebrafish fins, *cdkn1a* was strongly upregulated, *cdkn1ba* was not affected, and the third family member, *cdkn1ca*, was downregulated. This gene family provides another example of differential regulation depending on the cell and tissue type. CKI family members are well-connected in the SHOX-dependent gene regulatory network (Figure 5A), suggesting a role in chondrocyte proliferation, controlled by SHOX-dependent regulation. FGFR3 signaling induces *CDKN1A* expression (Su et al., 1997; Nakajima et al., 2003), so may directly link *SHOX* and *CDKN1A*.

The three members of the cytochrome P450 family 26, *CYP26A1*, *CYP26B1*, and *CYP26C1*, encode retinoic acid metabolizing enzymes involved in chondrogenesis (Pennimpede et al., 2010; Cunningham and Duester, 2015). *CYP26C1* is a genetic modifier of *SHOX* deficiency and downregulates *shox* expression in zebrafish (Montalbano et al., 2016). Moreover, *CYP26C1* variants cause isolated short stature in the absence of *SHOX* deficiency (Montalbano et al., 2018). Here, we show that *shox* knockdown significantly upregulates *cyp26c1* in zebrafish fins. This reciprocal regulation might maintain the delicate balance of retinoic acid levels during fin development. In addition, we observed dysregulation of *CYP26B1*/*cyp26b1* in NHDF cells and zebrafish fins, while *cyp26a1* was not affected by Shox. *Cyp26b1* regulates osteogenesis in the axial skeleton as indicated by the zebrafish *stocksteif* mutant (Spoorendonk et al., 2008). The detailed relationship between *CYP26B1* and *SHOX* deficiency so far remains unclear.

Our tissue-specific validation approach also indicates new roles for Shox during different embryonic developmental processes, including neuronal development and cardiogenesis, similar to/reminiscent of its paralog Shox2 (Yu et al., 2007; Vickerman et al., 2011; Liu et al., 2012; Ha and Dougherty, 2018; Hu et al., 2018). Shox2-regulated targets, such as *Bmp4* and *Nppb*, are involved in various biological processes such as heart and limb development (Yu et al., 2007; Puskaric et al., 2010; Aza-Carmona et al., 2014; Hoffmann et al., 2017).

The differential expression of *sox8a*, *bmp4*, *cyp26c1*, and *cdkn1a* in *shox*-deficient zebrafish hearts was clearly a Shox-specific effect since *shox2* expression was unaffected in this tissue, so did not have any indirect regulatory effects. While *shox* expression has previously been described in the zebrafish heart (Kenyon et al., 2011; Sawada et al., 2015), the role of shox during cardiogenesis remains unclear and requires further analysis in this model.

Shox-regulated gene expression in head tissue of zebrafish embryos also indicated a possible role for SHOX in brain development or neurodevelopmental disease. Microduplications at the pseudoautosomal SHOX locus have already been identified in patients with autism spectrum disorders (ASD) and related neurodevelopmental conditions (Tropeano et al., 2016). It is interesting to note that 17 of the 23 investigated genes are also associated with neuronal phenotypes including neurodegenerative diseases, intellectual disability/ASD, and psychiatric disorders (Supplementary Figure 2).

Evidence from both human and animal models has supported the notion that the genetic network involving *SHOX*, *FGFR3*, *NPPC*, among others, is an important regulator of growth. Mutations in these genes have not only been shown to cause rare skeletal malformations but also to contribute to the common forms of height (Rao et al., 1997; Ogata et al., 2000; Toydemir et al., 2006; Bocciardi et al., 2007; Tassano et al., 2013; Estrada et al., 2021). The target genes identified in this study add new members to this network which may contribute to rare skeletal forms or common forms of height and thus represent novel candidates for tall or short stature or other skeletal anomalies. Damaging mutations in these target genes may explain clinical phenotypes, e.g., short stature. As shown in Figure 5, some of the novel identified SHOX target genes have already been associated with a skeletal phenotype.

In conclusion, we have identified novel SHOX targets, many of which are consistent with known SHOX functions in limb development, and thereby provide novel insights into the genetic pathways leading to the clinical manifestation of *SHOX* deficiency. In addition, our tissue-specific approach suggests that some target genes may also play a role in other organs, such as heart and brain, indicating a pleiotropic role for SHOX in different phenotypic traits.

## Data Availability Statement

The datasets generated and analyzed for this study can be found in the public NCBI GEO database (GSE169732) and can be accessed *via* https://www.ncbi.nlm.nih.gov/geo/query/acc.cgi?acc=GSE169732.

## Ethics Statement

Ethical review and approval was not required for the animal study because only zebrafish embryos at early life stages (55 hpf) were used in this study.

## Author Contributions

SH and GR designed the study and experimental design and wrote the manuscript. Material preparation, acquisition, and data analysis were performed by SH, RR, SD, JG, DH, and SJ. All authors contributed to the article and approved the submitted version.

### Conflict of Interest

The authors declare that the research was conducted in the absence of any commercial or financial relationships that could be construed as a potential conflict of interest.

## Footnotes

^1^https://www.qiagenbioinformatics.com/products/ingenuity-pathway-analysis

^2^https://www.disgenet.org/home/
