# Supplementary material for: Identification and Tissue-Specific Characterization of Novel SHOX-Regulated Genes in Zebrafish Highlights SOX Family Members Among Other Genes
Source: Front Genet. 2021 May 27;12:688808. doi: 10.3389/fgene.2021.688808 (PMC8191631; doi:10.3389/fgene.2021.688808)
Supplement: Supplementary file 1 [file Data_Sheet_1.PDF]

Supplementary Material

Supplementary Figures

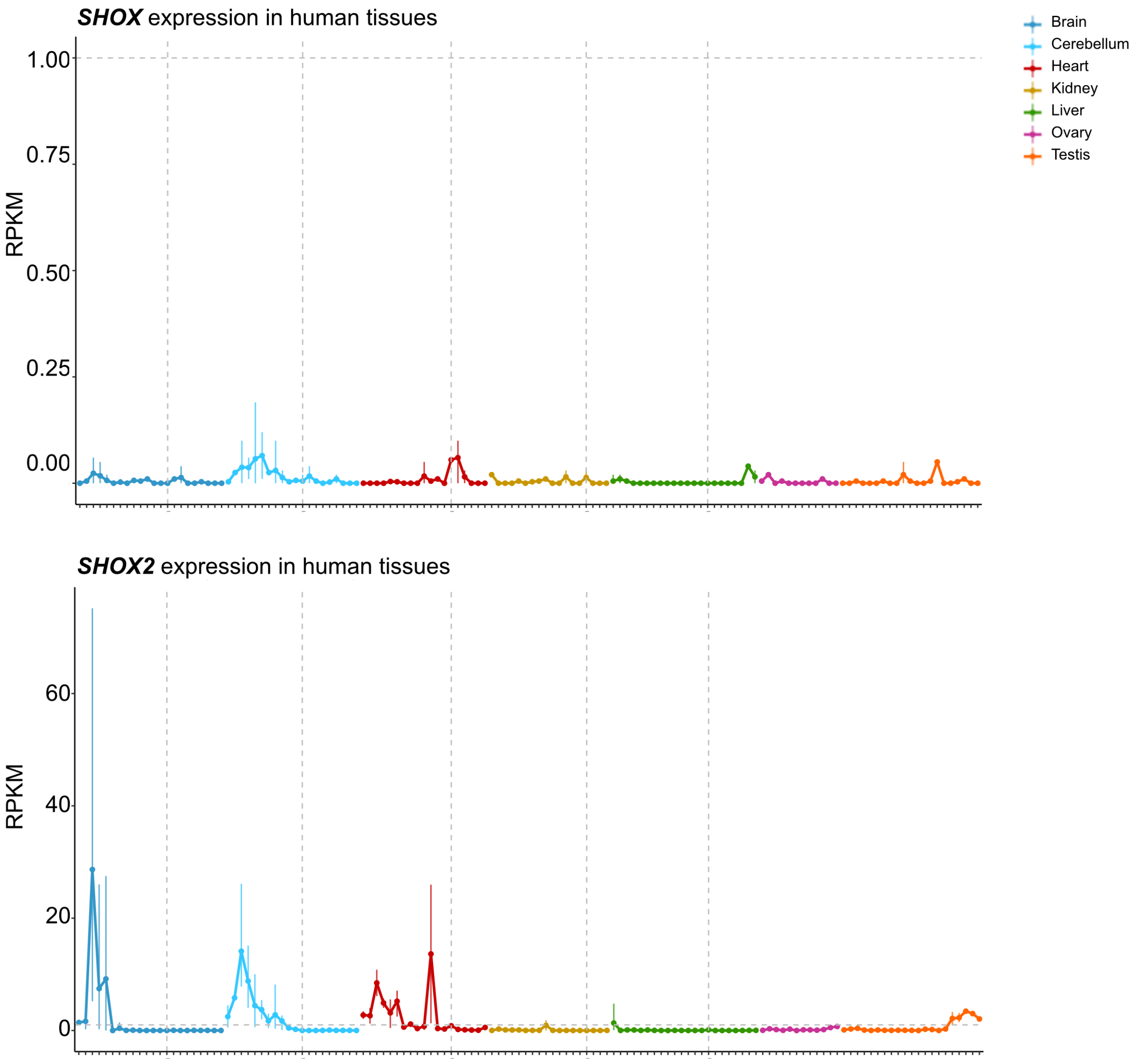

Supplementary Figure 1. ***SHOX* and *SHOX2* expression levels across different human tissues at different stages.** Each dot represents the median across replicates for a specific time point including prenatal stages (weekly between 4-20 weeks post conception), newborn, infant, toddler, school age, teenager, young adult (25-35 y), young mid age (36-45 y), older mid age (46-55 y), senior (56-65 y); the dahed vertical line indicates the newborn stage. Different tissues are presented in different colors. RPKM = Reads per kilobase of transcript per Million mapped reads. Data are exported from the 'Evo-devo mammalian organs app' (Cardoso-Moreira et al., 2019). Note that *SHOX* expression (upper diagram) is roughly 20 times lower compared to *SHOX2* expression (lower diagram).

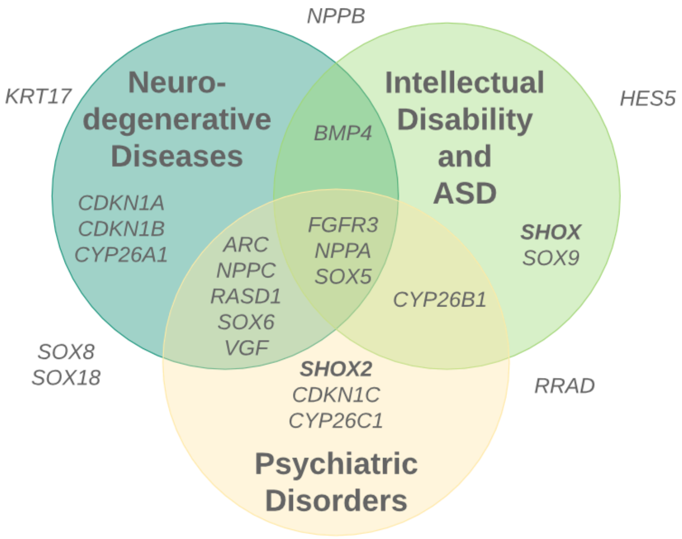

Supplementary Figure 2. Gene-disease association according to DisGeNET. Venn diagram showing associations between SHOX and 23 SHOX-regulated genes with respect to neuronal phenotypes based on the DisGeNET database (https://www.disgenet.org/home/) (Pinero et al., 2015).

Supplementary Tables

Gene	Accsession number	Target sequence (5' - 3')	Application
actb1	NM_131031.1	GTGCTTCTAAACAGAACTGTTGCCACCTTAAATGGCCTAGCAATGAGATTCAAACGAACGACCAACCTAAACTCTCGAACAGAACAAGATGACATCAGCA	nCounter Control probe
eef1a1l1	NM_131263.1	GAAGGCTGCCAAGACCAAGTGAATTTCCCTCAATCACACCGTTCCAAAGGTTGCGGCGTGTTCTTCCCAACCTCTTGGAATTTCTCTAAACCTGGGCACT	nCounter Control probe
rpl13a	NM_212784.1	AAGAGAAAGGAAAAGGCCAAGCTGCGCTATTCCAAGAAGAAAGTTGAGATGAAGCTGACTAAGCAGGCTGAAAAGAACGTTGAGAGCAAGATCGCAGTAT	nCounter Control probe
rps18	NM_173234.1	GTACAAAATCCCAGACTGGTTCCTGAACAGACAGAAGGACATAAAAGATGGGAAATACAGCCAGGTCCTTGCTAATGGTCTGGACAATAAACTGAGAGAA	nCounter Control probe
b2m	NM_001159768.1	TACTTTCGATATCAACTGCTGTTGTCCTGAATGCTGAAGGATTGTCTGCTTGGCTCTCTCGAATAAAACGGCCACAATGAGAGCACTCATCACTTTTGCA	nCounter Control probe
hsp90ab1	NM_131310.3	CTCACAGTCCGGCGACGAGATGACCTCCCTCACAGAATACGTCAGCCGTATGAAGGAGAACCAAAAGTCCATCTATTACATCACTGGTGAGAGCAAAGAC	nCounter Control probe
bmp4	NM_131342.2	GAGCCAACACCGTGAGAGGATTCCATCATGAAGAGCACCTGGAGGAGCTGCAGTCAGACGGCTCCCAGGAGACGCCTCTGCGATTCGTTTTTAATCTCAG	nCounter target probe
cdkn1a	NM_001128420.1	CACACACACAGATAAACTCTTGACCTGCATCTACCTTCATCTGTCTGGAGCTGCATTCGTCTCGTAGCAGCGTTATTGACAGAAAACGACCTTCAGGAAC	nCounter target probe
cdkn1ba	NM_001013268.1	CGGAAAAGTGGAATTACGACTTCGCCGAAAACCGACCGCTCGCGCCGGGCGACTACGAGTGGCAGGAGGTGGACGCCGACGAAGTGCCGGACTTTTACAC	nCounter target probe
cdkn1ca	NM_001002040.1	CAAAAAGAAAGAGGGTCGTGGAGTCGAAGCAGGATGAACGCAGTTACCTTCAAGCCGGCACATCCACGAGCATTGAAGTAACGCCACGTAAAACTATTAG	nCounter target probe
cyp26a1	NM_131146.2	GGTCAGAGAGGAGGTTCAGGAGAAGGTTGAAATGGGCATGTATACACCGGGAAAAGGCTTGAGCATGGAGCTGTTGGACCAGCTGAAGTACACTGGATGT	nCounter target probe
cyp26b1	NM_212666.1	CATCATCGGAGAAACATGCCACTGGTTCTTTCAGGGTGCAGGTTTCCATGCATCCAGAAGACAGAAGTATGGGAATGTATTCAAGACTCATCTACTGGGA	nCounter target probe
cyp26c1	NM_001029951.2	TGAATAATCTCTTCTCTCTTCCTATTGACACTCCTGTTAGCGGCCTGCGCAAGGGAATCAGAGCTCGTGAGATTCTGCACTCTGCCATGGAGAAGATCAT	nCounter target probe
fgfr3	NM_131606.2	TTTCGAGGATGCGGGGCAATACACTTGTCTGGCAGGGAACTCGATTGGCTATAACCATCACTCTGCTTGGCTTACAGTCTTACCAGCGGTGGAGATGGAG	nCounter target probe
her15.1	NM_182875.2	CAACAAGGAGAAGCACAAATTGCGAAAGCCAGTGGTGGAAAAGATGCGCAGAGATCGCATCAACAACTGCATCGAGCAGCTCAAGTCCATGCTGGAGAAA	nCounter target probe
krt17	NM_001083105.1	GCACGCACCCAGATGGGCGGACAAGTCAACGTAGAGGTTGACGCAGCACCACAGGAAGACCTGACAAAGATCATAGCTGATATCCGTGAGCACTATGAGG	nCounter target probe
nppa	NM_198800.3	CTGCTGCTCCTGGTTTGGCAGCAGACGGATGTACAAGCGCACACGTTGAGCAGACACAGCTCTGACAGCAACATGGCCAAGCTCAAGAGCTTGCTGCAGC	nCounter target probe

nppb	NM_001327776.1	TAAAGTTTCTCCTTCAACGACTTGAAGAGTCCATTCCAGCTCAAGACCAAACACCGGCGGAAAGAGAAGTAAAGGCGGCAAATATTGAAGAAACCCGAGC	nCounter target probe
nppc	NM_001109940.1	CTTGTGGACTTATTCTGACTCTCCTTTCAGTCAGCACGACAGAGACTAAACCTCTGACACAGGCTGAACAGAGGTCCCTCAGGGTGCTGCTGGGAGAGGA	nCounter target probe
rasd1	NM_200532.1	GGATACTTCAGGGAACCATCCATTCCCAGCTATGCGGAGACTGTCCATTCTGACCGGTGATGTGTTTATCCTGGTGTTCAGTTTGGACAACCGCGAGTCC	nCounter target probe
rrad	NM_199798.1	GACGTCCATCCTTCTGTATGACATATGGGAACAGGATAACAGCCAGTGGCTGCAGGATCAGTGCATGCGTATGGGAGATGCCTATATCATCGTGTACTCG	nCounter target probe
shox	NM_001126411.1	TTCTGGGCACCGCGAGCCACCTGGACGCGTGCAGGGTCGCTCCTTACGTGAATATGGGCGCACTCAGAATGCCATTTCAACAGGTTCAGGCTCAGTTGCA	nCounter target probe
shox2	NM_201196.1	TCAGAACCGAAGGGCTAAATGCAGAAAGCAGGAAAATCAGTTGCATAAAGGGGTTCTCATCGGAGCGGCGAGCCAGTTTGAAGCATGTCGCGTGGCTCCA	nCounter target probe
sox5	XM_005164766.4	TCTGAAGAGAGTAAGTTCACCCATGCAATGATGGACTTTGGCATCAGTGGCGATTCAGATGGAAGCCCAAGTGTGTCGGACTCGCGGATCTTCCGAGAGG	nCounter target probe
sox6	XM_021471907.1	TTTTCATGAATTCAAAATCAACTCACCAGTGATCTTTGGGAGCTGGGGATAAAGTCTTTCTGTTTATAACCCAAATGCTGAACATGGAGGGCTTTCTGCG	nCounter target probe
sox8a	NM_001284432.1	AATCTACAAGACGGAAGCGGGAATGAGAGCCATGCACCATCAGACCATTCATGGAGTTCAACCACACGGACCTCCAACGCCTCCAACCACACCGAAATCC	nCounter target probe
sox9a	NM_131643.1	TGAAGACGGCAGCGAACAGACCCACATCTCGCCCAACGCCATCTTCAAAGCGCTCCAGCAGGCGGACTCGCCCGCGTCCAGCATGGGAGAAGTGCACTCG	nCounter target probe
sox18	XM_017353772.2	CCCAACTACAAATACCGCCCTCGGAGGAAGAAACAACCCAAGAAGATGAAGCGAGTGGAGCCGGGTCTCCTTCTCCAAGGCTTGGCCCATGGAGGACCGG	nCounter target probe
vgf	XM_003198950.5	ATGTTATTCAGTCTCACAGTCAAGCCTGCAGCCTTTAGTCCAGTGACAACAGACAGAAACACAGTAAGTGAAAACTATGCAGCTGCCTCCTCTGCTCAGC	nCounter target probe

Supplementary Table 1. Sequences of nCounter probes.

Supplementary Table 2. List of differential expressed genes 6 hours upon *SHOX* overexpression.

Supplementary Table 3. List of differential expressed genes 12 hours upon *SHOX* overexpression.

Supplementary Table 4. List of differential expressed genes 24 hours upon *SHOX* overexpression.

Gene	ID	FoldChange	BeadSD	Timepoint
AIM2	004833.1	-3.635028051	24.47650876	12h
AQP1	198098.1	3.727868573	14.40162934	24h
ARC	015193.3	19.77923658	24.34592825	24h
ARC	015193.3	11.25888689	38.16658634	12h
ARL4	005738.2	-3.378721854	13.87773709	24h
ARL4	005738.2	-4.103333672	29.42443105	12h
ART5	053017.2	3.327852825	13.9645105	24h
BHLHB2	003670.1	3.066857167	13.47232622	24h
BTG2	006763.2	6.171374775	22.94823963	24h
BTG2	006763.2	4.159327088	23.5927742	12h
C1QTNF5	015645.2	3.995280319	14.94655589	24h
C9orf111	152286.2	3.398526985	12.83288607	24h
CBX4	003655.2	4.432306509	18.90491564	24h
CCL5	002985.2	-3.694696574	24.53694438	12h
CCL7	006273.2	-3.170138379	12.60595585	24h
CCL8	005623.2	-5.24357681	21.93414329	6h
CCL8	005623.2	-5.275549412	23.24038924	12h
CCL8	005623.2	-11.11711397	19.23781194	24h
CCRN4L	012118.2	-3.27045673	17.85896684	12h
CDKN1A	078467.1	5.441783006	20.20161696	24h
CDKN1C	000076.1	5.124274251	23.34225056	24h
CH25H	003956.2	-4.1882916	22.91735516	12h
CKB	001823.3	6.806497243	23.17218412	24h
CMYA1	194293.2	5.648575369	17.19551873	24h
CMYA1	194293.2	3.809411623	23.59413153	12h
CPEB3	014912.3	-3.243941366	24.37026784	12h
CPNE6	006032.2	3.847755532	15.08002577	24h
CRABP2	001878.2	4.695462007	20.14355315	24h
CXCL10	001565.1	-3.357058196	14.45688635	24h
CXCL10	001565.1	-3.633123807	21.72643457	12h
CXCL10	001565.1	-4.83406018	26.95392678	6h
CXCL11	005409.3	-3.83052818	14.96575621	12h

CXCL11	005409.3	-4.416886725	21.08525606	6h
CXCL11	005409.3	-4.442002072	20.57350452	24h
CYGB	134268.3	3.979541738	17.42294842	24h
CYP26B1	019885.2	17.32942768	23.66661554	24h
CYP26B1	019885.2	8.337945189	34.7866327	12h
DDX58	014314.2	-3.205182797	15.67057302	24h
DES	001927.3	5.984948723	17.7114306	24h
DES	001927.3	3.166408908	18.14779049	12h
DLK1	003836.4	4.539115165	18.64583063	24h
EEF1A2	001958.2	5.858812402	19.69441627	24h
EML2	012155.1	3.069882753	11.54056757	24h
EPHA2	004431.2	3.078790822	14.16022276	24h
FAM46B	052943.2	4.013128029	14.76042335	24h
FDXR	024417.1	3.004072177	19.15803229	24h
FGFR3	000142.2	10.51804315	26.51930799	24h
FGFR3	000142.2	7.123735029	36.42663822	12h
FLJ11000	018295.1	-3.035320445	14.78908489	24h
FLT4	002020.1	3.004546136	13.11779242	24h
FOS	005252.2	3.424016696	14.70102831	24h
FOSB	006732.1	3.063156419	15.30165429	24h
FZD10	007197.2	3.141271648	11.92472085	24h
FZD9	003508.2	3.277263746	16.32261659	24h
GADD45B	015675.1	7.643064067	28.34110354	24h
GADD45B	015675.1	3.201020062	23.93241504	12h
GALNT9	021808.2	3.55104685	12.73460491	24h
GBP1	002053.1	-3.137659209	20.75141385	12h
GBP1	002053.1	-3.261338552	15.36165924	6h
GBP1	002053.1	-5.253583345	17.91025806	24h
GBP4	052941.2	-3.267782816	19.98645662	12h
GBP4	052941.2	-3.511970822	20.49871793	6h
GBP4	052941.2	-4.682844059	16.86757287	24h
GDF15	004864.1	3.220426458	14.21052466	24h
GMPR	006877.2	-3.666676484	11.85324892	24h
GPR56	201525.1	6.729821703	20.35226688	24h
HAND1	004821.1	3.122134034	13.32782133	24h
HAPLN4	023002.1	3.645300301	19.66553821	24h
HBA1	000558.3	6.876603296	18.89109042	24h
HES5	001010926.1	36.13751742	23.52975434	24h
HES5	001010926.1	20.17635094	45.49834927	12h
HES6	018645.3	3.786882831	14.11217858	24h
HMOX1	002133.1	3.21243376	12.61512175	24h
HSPB8	014365.2	3.007771676	8.312306101	24h

IFI44L	006820.1	-4.28483911	16.90500658	24h
IFIT1	001548.2	-3.098517641	11.48473163	24h
IFIT1L	001010987.1	-3.129319003	18.57875074	12h
IFIT3	001549.2	-4.013733699	18.35078088	24h
IFNB1	002176.2	-4.807647677	18.82686397	24h
IFNB1	002176.2	-5.029595911	26.55623247	6h
IFNB1	002176.2	-8.597752841	13.41149791	12h
IL8	000584.2	-3.081971873	18.31674504	6h
INA	032727.2	3.83455038	13.8627148	24h
INDO	002164.3	-4.478807409	20.99638291	6h
INDO	002164.3	-4.853005195	15.03427472	12h
INHBB	002193.1	3.778661734	16.7627224	24h
JUND	005354.2	3.006685136	19.0471416	24h
KCNF1	002236.4	6.425251247	22.95978095	24h
KCNN4	002250.2	3.713098703	17.15154778	12h
KCNN4	002250.2	3.359224764	10.90802242	24h
KIF1A	004321.4	5.17634924	24.18343343	24h
KRT17	000422.1	9.911624665	25.9720412	24h
KRT17	000422.1	5.12856248	29.41648747	12h
KRT18	199187.1	4.254117771	14.24925238	24h
LFNG	002304.1	4.466811237	16.49679851	24h
LFNG	002304.1	3.662064937	17.65110495	12h
LOC129607	207315.1	-3.437383781	14.04412518	24h
LOC144501	182507.1	3.450372265	18.23277423	24h
LOC402573	001004323.1	3.175519171	11.06229306	24h
LRRN3	018334.3	-3.827311107	26.03985977	12h
MSX1	002448.1	3.109982806	15.84035095	24h
MYEOV	138768.2	6.365025753	21.06670918	24h
NCOA7	181782.2	-3.548123911	14.04704433	24h
NFKBIZ	001005474.1	-3.8149287	27.47187797	12h
NPPB	002521.1	12.18828064	26.84057456	24h
OAS1	002534.2	-3.088070831	17.58645005	24h
OAS1	001032409.1	-3.446215725	18.34733853	24h
PDGFRB	002609.3	3.82578529	15.69643966	24h
PHLDA2	003311.3	3.061749744	14.72865076	24h
PIM3	001001852.2	4.223112232	21.04866993	24h
PRIC285	033405.2	-3.512120176	14.34600809	24h
PRSS8	002773.2	6.335537924	19.82409549	24h
PTGER4	000958.2	-3.105157765	14.8664244	12h
RAC2	002872.3	6.27793421	24.17275602	24h
RAP1GA1	002885.1	5.236577654	20.41462756	24h
RASD1	016084.3	15.18306472	27.77034967	24h
RASD1	016084.3	4.396514974	26.91163493	12h

RGS16	002928.2	3.318642558	21.968034	12h
RRAD	004165.1	17.67616362	23.38822132	24h
RRAD	004165.1	11.52308227	37.12487287	12h
RUNX3	004350.1	3.570351936	13.38433268	24h
SAMD9L	152703.2	-3.32219039	13.49694463	24h
SBSN	198538.1	3.95922006	17.93747201	24h
SDC1	002997.4	3.061228317	17.04076144	24h
SLC9A3R1	004252.1	3.198586709	10.0899394	24h
SNF1LK	173354.2	5.704903582	17.66900355	24h
SNF1LK	173354.2	4.067849184	20.73960115	12h
SOX18	018419.2	8.959198294	21.82659099	24h
SOX18	018419.2	6.448122491	38.27037908	12h
SOX8	014587.2	44.58511567	31.66231219	24h
SOX8	014587.2	16.23846325	37.66333021	12h
SOX8	014587.2	6.410477094	30.9819678	6h
SP110	004510.2	-3.101841032	16.11363579	24h
SRPK2	182691.1	-3.082375391	17.03222762	12h
STMN3	015894.2	5.998370727	20.27218964	24h
TNF	000594.2	6.243408468	19.86632815	24h
TNF	000594.2	4.22271755	24.83632638	12h
TNFRSF25	148973.1	3.282902944	16.91207636	24h
TNFSF10	003810.2	-3.255737195	17.61136366	12h
TNFSF9	003811.2	5.480724089	16.30960974	24h
TNFSF9	003811.2	3.490297139	21.00267515	12h
TSPAN9	006675.3	5.532926959	21.26607147	24h
TUBB2B	178012.3	3.848625881	11.51842069	24h
VGF	003378.2	22.03990909	34.84279633	24h
VGF	003378.2	7.395371664	31.76914028	12h

Supplementary Table 5. Microarray data of *SHOX* overexpression NHDF cells after 6, 12 and 24 hours (h). Combined list of differentially expressed genes with a more than 3-fold change in expression level.

Selected SHOX Targets, Interacting Proteins, and Modulators of Activity

Category	Gene	Interactions with SHOX	Reference
Transcriptional direct targets	*FGFR3*	Regulated by SHOX in human cells and chicken micro mass	Decker et al., 2011
	NPPB	Regulated by SHOX in human cells and growth plate	Marchini et al., 2007
Upstream regulators	*BMP4*	Regulates Shox expression negatively in chicken embryos	Tiecke et al., 2006
Physical interacting proteins	*SHOX2*	Forms heterodimers with SHOX in human cells	Aza-Carmona et al., 2014
	SOX5	Forms a complex with SHOX together with SOX6 and SOX9 in human cells and growth plate	Aza-Carmona et al., 2011
	SOX6	Forms a complex with SHOX together with SOX5 and SOX9 in human cells and growth plate	Aza-Carmona et al., 2011
	SOX9	Forms a complex with SHOX together with SOX5 and SOX6 in human cells and growth plate	Aza-Carmona et al., 2011
Cellular mediators	*CDKN1A*	Up-regulated in SHOX-expressing cells; involved in SHOX-induced cell cycle arrest in human cells	Marchini et al., 2004
	CDKN1B	Up-regulated in SHOX-expressing cells; involved in SHOX-induced cell cycle arrest in human cells	Marchini et al., 2004
	CYP26C1	Genetic modifier of SHOX deficiency in humans and zebrafish	Montalbano et al., 2016

Supplementary Table 6. Published SHOX-associated genes adapted from (Marchini et al., 2016).

	Diseases or Functions Annotation	*p*-value
1	Differentiation of oligodendrocytes	7.78E-13
2	Development of chondrocytes	2.19E-12
3	Apoptosis	2.53E-10
4	Differentiation of heart cells	8.17E-10
5	Development of internal genitalia	1.03E-09
6	Cartilage development	1.13E-09
7	Growth of connective tissue	1.47E-09
8	Differentiation of oligodendrocyte precursor cells	1.53E-09
9	Differentiation of nervous system	1.58E-09
10	Expression of RNA	1.95E-09
11	Formation of cartilage tissue	2.85E-09
12	Differentiation of chondrocytes	3.60E-09
13	Development of connective tissue cells	7.27E-09
14	Transcription of RNA	8.12E-09
15	Transcription	8.89E-09
16	Catabolism of tretinoin	1.02E-08
17	Proliferation of connective tissue cells	1.05E-08
18	Differentiation of central nervous system cells	1.47E-08
19	Development of central nervous system	2.57E-08
20	Quantity of epithelial cells	2.93E-08
21	Limb development	2.94E-08
22	Production of cells	3.12E-08
23	Morphology of bone	5.30E-08
24	Receptor guanylyl cyclase signaling pathway	5.71E-08
25	Development of genital organ	7.23E-08

Supplementary Table 7. List of Top 25 annotated pathways relevant to disease or function based on IPA analysis.

Supplementary References

Cardoso-Moreira, M., Halbert, J., Valloton, D., Velten, B., Chen, C., Shao, Y., Liechti, A., Ascencao, K., Rummel, C., Ovchinnikova, S., Mazin, P.V., Xenarios, I., Harshman, K., Mort, M., Cooper, D.N., Sandi, C., Soares, M.J., Ferreira, P.G., Afonso, S., Carneiro, M., Turner, J.M.A., Vandeberg, J.L., Fallahshahroudi, A., Jensen, P., Behr, R., Lisgo, S., Lindsay, S., Khaitovich, P., Huber, W., Baker, J., Anders, S., Zhang, Y.E., and Kaessmann, H. Gene expression across mammalian organ development. Nature (2019) 571**:**505-509.

Marchini, A., Ogata, T., and Rappold, G.A. A Track Record on SHOX: From Basic Research to Complex Models and Therapy. Endocr Rev (2016) 37**:**417-448.

Pinero, J., Queralt-Rosinach, N., Bravo, A., Deu-Pons, J., Bauer-Mehren, A., Baron, M., Sanz, F., and Furlong, L.I. DisGeNET: a discovery platform for the dynamical exploration of human diseases and their genes. Database (Oxford) (2015) 2015**:**bav028.
